# microRNAs as Potential Biomarkers in Adrenocortical Cancer: Progress and Challenges

**DOI:** 10.3389/fendo.2015.00195

**Published:** 2016-01-20

**Authors:** Nadia Cherradi

**Affiliations:** ^1^U1036, Institut National de la Santé et de la Recherche Médicale, Grenoble, France; ^2^Biologie du Cancer et de l’Infection, Commissariat à l’Energie Atomique, Institut de Recherches en Technologies et Sciences pour le Vivant, Grenoble, France; ^3^Laboratoire BCI, Université Grenoble-Alpes, Grenoble, France

**Keywords:** adrenocortical carcinoma, circulating miRNA, biomarker, diagnosis, prognosis, therapeutic targets

## Abstract

Adrenocortical carcinoma (ACC) is a rare malignancy with poor prognosis and limited therapeutic options. Over the last decade, pan-genomic analyses of genetic and epigenetic alterations and genome-wide expression profile studies allowed major advances in the understanding of the molecular genetics of ACC. Besides the well-known dysfunctional molecular pathways in adrenocortical tumors, such as the IGF2 pathway, the Wnt pathway, and TP53, high-throughput technologies enabled a more comprehensive genomic characterization of adrenocortical cancer. Integration of expression profile data with exome sequencing, SNP array analysis, methylation, and microRNA (miRNA) profiling led to the identification of subgroups of malignant tumors with distinct molecular alterations and clinical outcomes. miRNAs post-transcriptionally silence their target gene expression either by degrading mRNA or by inhibiting translation. Although our knowledge of the contribution of deregulated miRNAs to the pathogenesis of ACC is still in its infancy, recent studies support their relevance in gene expression alterations in these tumors. Some miRNAs have been shown to carry potential diagnostic and prognostic values, while others may be good candidates for therapeutic interventions. With the emergence of disease-specific blood-borne miRNAs signatures, analyses of small cohorts of patients with ACC suggest that circulating miRNAs represent promising non-invasive biomarkers of malignancy or recurrence. However, some technical challenges still remain, and most of the miRNAs reported in the literature have not yet been validated in sufficiently powered and longitudinal studies. In this review, we discuss the current knowledge regarding the deregulation of tumor-associated and circulating miRNAs in ACC patients, while emphasizing their potential significance in pathogenic pathways in light of recent insights into the role of miRNAs in shaping the tumor microenvironment.

## Introduction

Adrenocortical cancer is a rare and aggressive malignancy (with an incidence of 0.7–2.0 cases per million per year). Patients with adrenocortical carcinoma (ACC) generally have a poor prognosis, with a 5-year survival rate ranging from 15 to 30% in most series (1). Most patients present with advanced disease or develop local recurrence and distant metastasis post-operatively. In addition, despite the development of systematic classification algorithms (2), it is sometimes challenging to discriminate malignant tumors from their benign counterparts. Currently, the only curative approach to localized ACC is complete tumor resection. Adjuvant mitotane therapy has been shown to improve recurrence-free survival following complete surgical resection (3).Nevertheless, this adrenolytic drug causes significant toxicity and adverse effects (4). Adjuvant radiation therapy showed no advantage in terms of overall survival (5–7). In metastatic disease, mitotane has produced very limited objective response (8) and remains the only drug approved by the U.S Food and Drug Administration (FDA) and the European Medicine Executive Agency (EMEA). The First International Randomized trial in locally advanced and Metastatic Adrenocortical Carcinoma Treatment (FIRM-ACT) reported that the combination of mitotane with the chemotherapeutic agents etoposide–cisplatin–doxorubicin was associated with a better progression-free survival than the association of mitotane with streptozotocin (9). However, the overall survival did not differ between both arms. In light of these therapeutic failures, molecular targeted therapies have been tested in patients with metastatic ACC. These approaches include epidermal growth factor receptor (EGFR) inhibitors (10), anti-vascular growth factor antibodies (bevacizumab) (11), and tyrosine kinase inhibitors (Sorafenib, Sunitinib) (12, 13). More recently, drugs targeting the IGF2/IGF-1R signaling pathway have been evaluated (14–16). All these targeted therapies yielded disappointing results in terms of progression-free and overall survival. In this context, there is a critical need for additional tools to improve diagnosis and prognosis and to explore new targeted therapies. Over the last decade, gene expression profiling using DNA microarray analysis has emerged as a useful technique for tumor classification (17–25). Increased *IGF2* expression was identified in most studies as one of the most dominant transcriptional change specifically present in ACC relative to benign tumors (adenomas, ACA) and normal adrenal (NA). More recently, an integrated genomic characterization of ACC, combining exome sequencing, SNP arrays, DNA methylation analysis, mRNA expression arrays, and microRNAs (miRNAs) sequencing provided a comprehensive overview of known drivers genes (*CTNNB1, TP53, CDKN2A, RB1*, and *MEN1*) and newly identified altered pathways (*ZNRF3, DAXX, TERT*, and *MED12*) in ACC (26). It appeared that aggressive and non-aggressive ACC are two distinct diseases with specific gene signatures and alterations.

**Figure 1:**
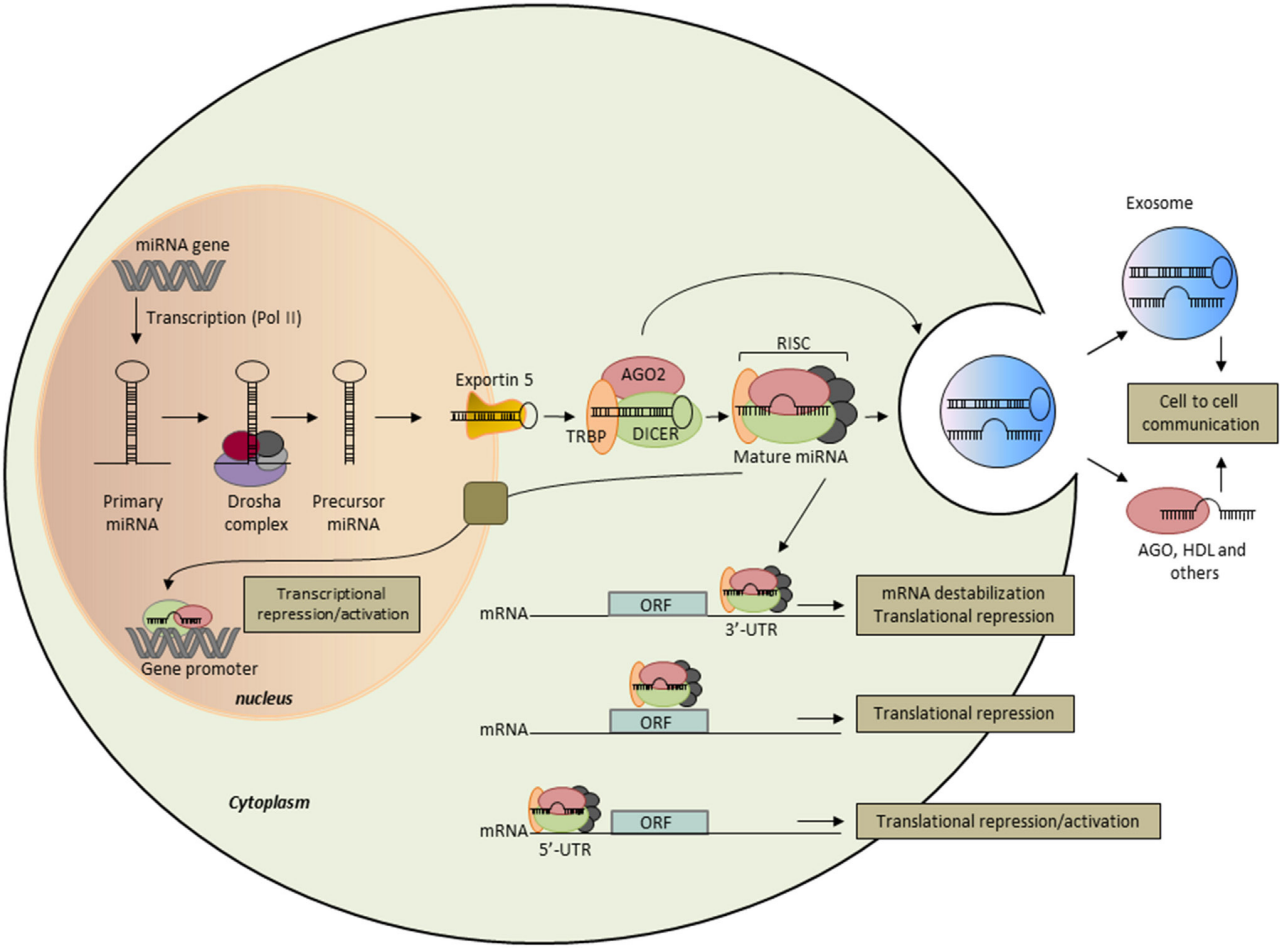
**Biogenesis and function of microRNAs**. miRNA genes are transcribed as primary miRNAs (Pri-miRNA) by polymerase II (Pol II) in the nucleus. Pri-miRNAs are cleaved by the RNAse III endonuclease DROSHA and its proteins partners (DROSHA complex) to produce the 60- to 70-nt stem–loop precursor miRNAs (pre-miRNAs). The pre-miRNAs are then exported to the cytoplasm by exportin 5 and further processed by DICER1, a type III RNAse that produces the 22-nt mature miRNAs. One strand of the mature miRNA is selectively loaded into the miRNA-induced silencing complex (RISC), which contains DICER1, Argonaute (AGO) proteins, and the transactivation-responsive RNA-binding protein (TRBP). In the cytoplasm, mature miRNAs bind essentially to the 3′-UTR of the target mRNA and repress its expression through both translational repression and mRNA destabilization. Some miRNAs have been shown to bind to the open reading frame (ORF) and the 5′-UTR of the target mRNA, and to activate or repress its translation efficiency. In the nucleus, miRNAs were shown to bind to gene promoter to regulate gene expression. miRNAs are also released into the extracellular space and are possibly involved in intercellular communication when transferred to target cells. Extracellular miRNAs are encapsulated within microvesicles, such as exosomes, or bound to RNA-binding proteins, such as Ago2, or lipoproteins, such as HDL.

In mammals, miRNAs were discovered a decade ago as an abundant class of small non-coding RNA (18–24 nt in length) that silence their target genes at the post-transcriptional level, either by degrading mRNA or by inhibiting translation (27). Comparative sequence analyses combined to computational methods predict that miRNAs could regulate the expression of more than 50–60% of human coding genes (28). The latest version of miRBase (Release 21, June 2014) has annotated over 2000 miRNA sequences in the human genome and novel ones are reported at a constant rate as more tissues are sequenced to greater depth. The biogenesis of miRNAs consists of multiple steps (29) (Figure 1). The primary miRNA (pri-miRNA) transcript is transcribed by RNA polymerase II, then cleaved by the complex Drosha to release a hairpin-structured miRNA precursor (pre-miRNA) in the nucleus. Pre-miRNA is transported from the nucleus to the cytoplasm by exportin-5-Ran-GTP-dependent double-stranded (ds) RNA-binding protein then processed into a short ds miRNA duplex by the ribonuclease III Dicer. Following unwinding of the duplex, the resultant guide strand mature miRNA is preferentially assembled into the RNA-induced silencing complex (RISC) composed of Dicer, Argonaute 2 (Ago2), and the dsRNA-binding protein TRBP. The association of the miRNA–RISC complex to complementary sequences in the 3′-untranslated region (3′-UTR) of target mRNA leads to inhibition of protein translation or degradation of the mRNA. More recently, it has been shown that miRNAs may target protein coding as well as 5′-UTR regions (30). An additional layer of complexity has been added since miRNAs were demonstrated to modulate gene expression at transcriptional level through their interaction with the transcription machinery or promoter sequences (31). Many miRNAs exhibit tissue-specific pattern of expression, suggesting that they play critical role in tissue and organ development, function, and maintenance. Each miRNA can control hundreds of genes and a single transcript harbors binding sites for several miRNAs. Due to their potential multi-target actions, it is not surprising that miRNAs regulate a plethora of basic biological mechanisms, such as cell cycle control, apoptosis, cell proliferation, differentiation, migration, and invasion, that impact systems biology in cancer.

A link between miRNAs and cancer was brought by the seminal observation of Croce’s group who reported that miR-15 and miR-16, two miRNAs located in chromosome 13 (13q14) are frequently deleted in chronic lymphocytic leukemia (CLL) and function as tumor suppressors (32, 33). Since then, miRNAs have been studied most intensively in the field of cancer research and growing evidence suggests that altered miRNA expression is involved in the pathogenesis of cancers. The causes of the deregulation of miRNA expression in cancer cells are only partially elucidated. So far, at least three different mechanisms that could function independently or together have been described. The first one is that half of the known miRNAs are located in regions of chromosomal instability associated with cancer, including regions of loss of heterozygosity (LOH), regions of amplification, and fragile sites (34). The second mechanism involves epigenetic regulation of miRNA expression. DNA hypomethylation, CpG island hypermethylation and histone-modification losses have been shown to also affect miRNA expression (35). For example, histone deacetylase inhibition in breast cancer cells was followed by the extensive alteration of miRNA levels (36). The third mechanism is abnormalities in miRNA-processing genes and proteins (35). As the machinery involved in the biogenesis and maturation of miRNAs involve multiple protein complexes, one can anticipate that alterations of these proteins should have dramatic effects on miRNA expression. An analysis of gene expression in a wide range of primary tumors revealed that the downregulation of miRNAs observed in cancer was due to a failure at the Drosha processing step although the mechanisms underlying these dysregulations were not elucidated in this study (37). Interestingly, it was subsequently reported that p53 promotes the Drosha-mediated processing of certain miRNAs with growth-suppressive function (38). Consequently, p53 gene mutations may lead to decreased processing of pri-miRNAs by Drosha and decreased levels of mature miRNAs in cancer cells.

**Figure 2:**
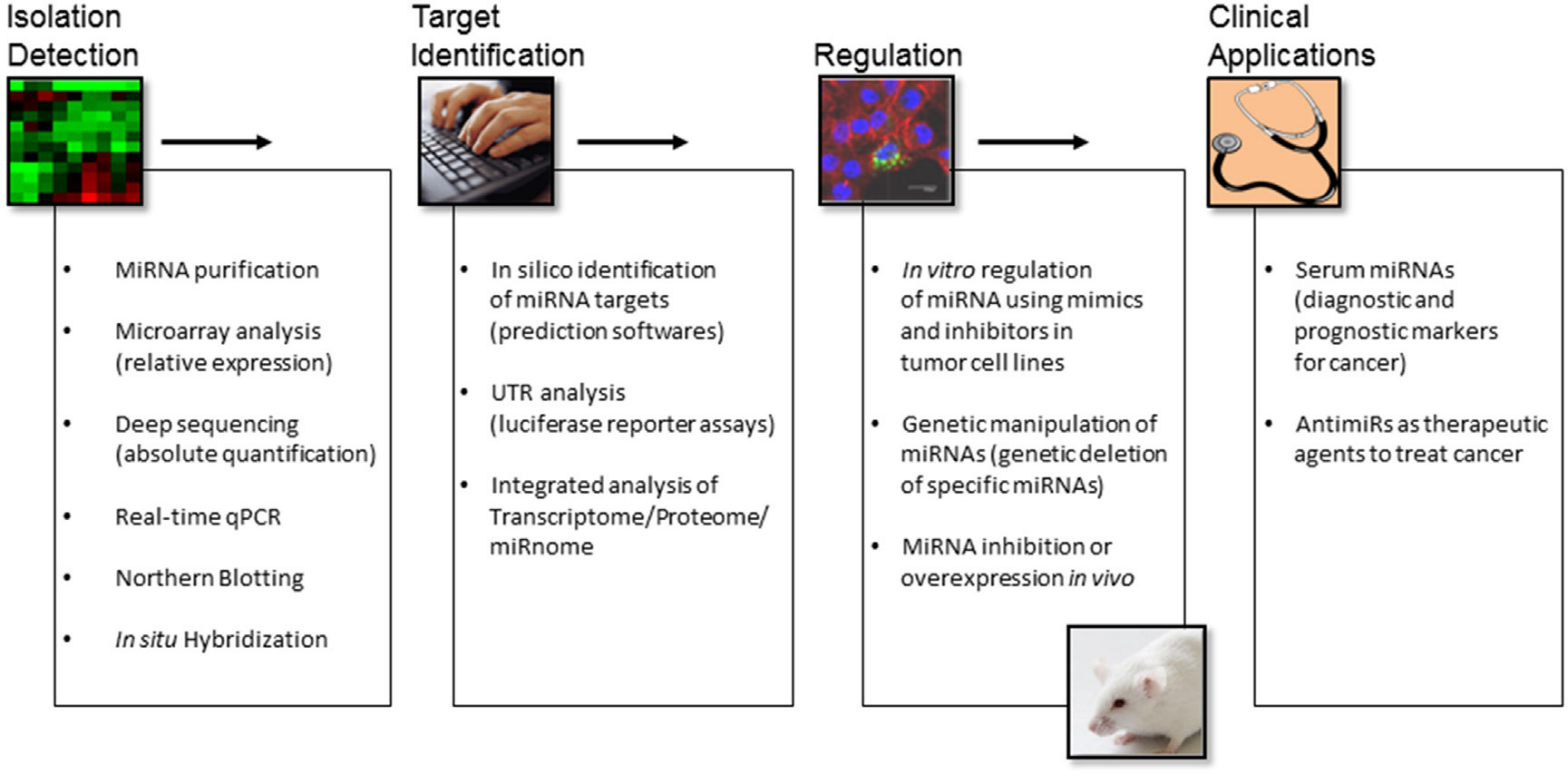
**An overview of miRNA research tools for isolation, detection, target determination, regulation, and clinical applications**.

The abnormal levels of miRNAs in tumors have important pathogenic consequences: miRNAs that are overexpressed in tumors contribute to oncogenesis by downregulating tumor suppressor genes, whereas underexpressed miRNAs contribute to oncogene expression. However, certain miRNAs may function as tumor suppressors or oncogenes depending on the cell-type-specific microenvironment, which may provide a different repertoire of available target genes. Identification of specific miRNA expression patterns for different tumor histological types is a useful complement for the classification of tumors that otherwise cannot be accurately diagnosed by classical morphology-based methods. Interestingly, Lu et al. showed that the expression levels of 217 miRNAs classified poorly differentiated tumors better than information obtained from microarray analysis of about 16,000 mRNAs (39). The diagnostic power of these miRNA profiles strongly support the key role of miRNAs in developing and maintaining cellular fates. On the other hand, the potential role of miRNAs as prognostic and predictive biomarkers in cancer patient management has been suggested by numerous studies. One of the most recent exciting findings is that cell-derived miRNAs exist with remarkable stability in various types of body fluids, including blood. Circulating miRNAs are encapsulated in microparticles (microvesicles, exosomes, and apoptotic bodies) or associated with RNA-binding proteins (Ago2), or lipoprotein complexes (high-density lipoproteins) (40). Although previously considered to be cellular waste products, recent studies have demonstrated that exosomes are “bioactive vesicles” that promote intercellular communication by shuttling molecules between cells (41). Importantly, specific circulating miRNA concentrations correlate with the development and progression of cancer (42). Circulating miRNAs fulfill several properties of non-invasive and good biomarkers, such as availability in various body fluids, sequence conservation between human and various preclinical models, and available sensitive technologies for their quantification. The technologies used to measure miRNA levels include microarray, next-generation sequencing (NGS), and reverse-transcriptase quantitative Polymerase Chain Reaction (RT-qPCR). Both microarray and NGS-based platforms are suitable for screening and discovery purposes, but qPCR remains the top choice for validation and clinical tests. Regarding miRNA target identification, it is worth mentioning that this task is largely limited due to the imperfect complementarity of miRNAs and target transcripts. Canonical miRNA targeting is characterized by the perfect pairing of the miRNA’s seed sequence, usually comprising 2–7 nt at the 5′-end of the miRNA, which is accompanied by base pairing at the miRNA’s 3′-end (43). However, non-canonical targeting that lacks continuous seed pairing, but relies on increased complementarity toward the miRNA’s center and/or 3′-end has been reported (44). Notably, only canonical targeting of miRNAs can be predicted by available *in silico* tools. Although these algorithms provide useful insights in some cases, these approaches remain challenging. Integrated transcriptome, proteome, and miRnome analyses to identify functional mRNA targets of miRNAs with altered expression may add valuable information on changes in gene regulation. On the other hand, because miRNAs affect the expression of multiple genes and thereby tune multiple steps in oncogenic pathways, they represent interesting therapeutic targets. The potential for using miRNAs in cancer therapy is now being explored thanks to the new advances in delivery of miRNA inhibitors or miRNA mimics (45). An overview of miRNA research tools for isolation, detection, target determination, regulation, and clinical applications is presented in Figure 2. In the present review, we summarize the findings related to miRNA deregulation in ACC with a focus on specific miRNA members and discuss their intrinsic merits and challenges for their use as diagnostic and prognostic biomarkers as well as potential therapeutic targets in ACC.

## Global Changes in miRNA Expression and in miRNA Biogenic Machinery in Adrenocortical Cancer

Due to the rarity of adrenocortical cancer, our understanding of the relevance of miRNAs in the pathogenesis of this disease is still in its infancy. While the role of miRNAs in the development and progression of a most common cancer, such as lung cancer, has reached several hundred publications in Medline, about 30 studies have been published on miRNAs in ACC. The number of validated target genes for deregulated miRNAs in ACC remains very limited (46–50). Thus, in-depth analyses of the mechanisms underlying miRNA deregulations in ACC and their role in aberrant gene expression remain to be conducted. Network algorithms could be effective in testing for potential associations between miRNA clusters and gene expression alterations. For example, integration of certain dysregulated miRNAs into gene networks established from ACC omics datasets revealed their potential role in specific signaling pathways in adrenocortical cancer (51). A new intricate dimension has been added to miRNA regulation since it was discovered that miRNAs are themselves targeted by regulatory RNA species (52). Recent studies identified competing endogenous RNAs (ceRNAs) or natural miRNA sponges that titrate miRNA availability (53). Such miRNA sponges bind miRNAs and competitively sequester them from their physiologically relevant targets. This class of sponges includes endogenously transcribed pseudogenes, long non-coding RNAs (lncRNA), and recently discovered circular RNAs. They may act in large complex networks in conjunction with miRNAs to regulate protein levels. Interestingly, lncRNA dysregulation has been recently reported in adrenocortical tumors (54). The impact of lncRNAs on miRNA expression and function in ACC awaits further investigations.

Besides chromosomal alterations, major dysfunctional pathways in ACC, such as somatic mutations of the tumor suppressor gene TP53, overexpression of IGF2, and activation of the Wnt/β catenin signaling pathway, are likely to impact miRNA expression. Global alterations in the expression of miRNAs in ACC compared to ACA or to normal adrenocortical tissue (NA) have been reported in nine retrospective studies conducted in adult patients (Table 1). Only one study has assessed the expression of a set of miRNAs in childhood adrenocortical tumors (46). Microarrays and qPCR have been the main strategies applied to investigate the link between miRNAs and adrenocortical tumors (Table 1). More recently, NGS brought a new informative landscape on miRNA expression in adrenocortical cancer (26) (discussed below). Despite this rapid progress, many challenges related to miRNA biomarker development for ACC include variations in sample collection and processing, in quantification methods and normalization controls as well as in data analysis. Independent studies using small cohorts and different miRNA detection platforms have often reported poorly overlapping results. So far, none of the miRNAs identified as potential biomarkers for ACC have been validated in appropriately powered clinical studies. International collaborative studies using large cohorts, standardized procedures, and consensual rules for statistical analyses would enable to establish robust miRNA signatures. In this context, the use of RNA sequencing would enable to address different questions that remain unanswered by RT-qPCR or microarray approaches, such as the detection of single nucleotide variants and copy number as well as the discovery of novel miRNAs. In addition, RNA sequencing is not biased by thermodynamics, a drawback of qPCR and microarrays platforms. However, potential limitations of NGS include the high cost and the computational infrastructure needed for data analysis and interpretation. A detailed description of miRNAs that were found deregulated in ACC has been provided in previous reviews (55, 56). Here, we will focus on miRNAs that were consistently reported as differentially expressed in tumor tissue and serum or plasma among different studies.

**Table 1:** **Significantly deregulated microRNAs in adrenocortical cancer**.

Validated miRNA in ACC compared to ACA or NA	Sample type and method	Cohort composition	Signature	Reference
**miR-483-5p**	Microarray/RT-qPCR	22 ACC, 27 ACA, 6 NA	↑^c^	Soon et al. (24, 57)
	Microarray/RT-qPCR	10 ACC, 26 ACA, 21 NA	↑	Patterson et al. (58)
	Microarray/RT-qPCR	25 ACC, 43 ACA, 10 NA	↑	Ozata et al. (47)
	RT-qPCR	18 ACC^a^, 10 ACA, 3 NA	↑	Chabre et al. (59)
	RT-qPCR	51 ACC	↑	Duregon et al. (60)
	NGS	45 ACC^a^, 3 NA	↑	Assie et al. (26)
	Microarray/RT-qPCR	17 ACC, 29 ACA	↑	Feinmesser et al. (61)
**miR-483-3p**	Microarray/RT-qPCR	10 ACC, 26 ACA, 21 NA	↑	Patterson et al. (58)
	Microarray/RT-qPCR	25 ACC, 43 ACA, 10 NA	↑	Ozata et al. (47)
	RT-qPCR	51 ACC	↑	Duregon et al. (60)
	NGS	45 ACC^a^, 3 NA	↑	Assie et al. (26)
	Microarray/RT-qPCR	17 ACC, 29 ACA	↑	Feinmesser et al. (61)
**miR-210**	TLDA	7ACC, 19 ACA, 10 NA	↑	Tombol et al. (25, 62)
	Microarray/RT-qPCR	25 ACC, 43 ACA, 10 NA	↑	Ozata et al. (47)
	RT-qPCR	51 ACC	↑^b^,^c^	Duregon et al. (60)
	NGS	45 ACC^a^, 3 NA	↑^b^	Assie et al. (26)
	Microarray/RT-qPCR	17 ACC, 29 ACA	↑	Feinmesser et al. (61)
**miR-503**	TLDA	7ACC, 19 ACA, 10 NA	↑	Tombol et al. (25, 62)
	Microarray	25 ACC, 43 ACA, 10 NA	↑^c^	Ozata et al. (47)
	NGS	45 ACC^a^, 3 NA	↑^b^	Assie et al. (26)
	Microarray/RT-qPCR	17 ACC, 29 ACA	↑	Feinmesser et al. (61)
**miR-184**	TLDA	7ACC, 19 ACA, 10 NA	↑	Tombol et al. (25, 62)
	NGS	45 ACC^a^, 3 NA	↑^b^	Assie et al. (26)
miR-21	Microarray/RT-qPCR	25 ACC, 43 ACA, 10 NA	↑	Ozata et al. (47)
miR-1202	*id*.	*id*.	↑^c^	*id*.
miR-1275	*id*.	*id*.	↑^c^	*id*.
miR-139-5p	Microarray/RT-qPCR	12 ACC^a^, 6 ACA + validation cohort (18 ACC^a^, 10 ACA, 3 NA)	↑^b^	Chabre et al. (59)
	NGS	45 ACC^a^, 3 NA	↑^b^	Assie et al. (26)
miR-376a	Microarray/RT-qPCR	12 ACC^a^, 6 ACA + validation cohort (18 ACC^a^, 10 ACA, 3 NA)	↑^b^	Chabre et al. (59)
	NGS	45 ACC^a^, 3 NA	↑^b^	Assie et al. (26)
**miR-195**	Microarray/RT-qPCR	22 ACC, 27 ACA, 6 NA	↓^c^	Soon et al. (24, 57)
	Microarray/RT-qPCR	10 ACC, 26 ACA, 21 NA	↓	Patterson et al. (58)
	Microarray/RT-qPCR	25 ACC, 43 ACA, 10 NA	↓	Ozata et al. (47)
	Microarray/RT-qPCR	12 ACC^a^, 6 ACA + validation cohort (18 ACC^a^, 10 ACA, 3 NA)	↓	Chabre et al. (59)
	NGS	45 ACC^a^, 3 NA	↓	Assie et al. (26)
	Microarray/RT-qPCR	17 ACC, 29 ACA	↓	Feinmesser et al. (61)
**miR-335**	Microarray/RT-qPCR	22 ACC, 27 ACA, 6 NA	↓	Soon et al. (24, 57)
	TLDA	4 ACC, 9 ACA, 4 NA + validation cohort (*n* = 15)	↓	Schmitz et al. (63)
	Microarray/RT-qPCR	12 ACC^a^, 6 ACA + validation cohort (18 ACC^a^, 10 ACA, 3 NA)	↓	Chabre et al. (59)
	NGS	45 ACC^a^, 3 NA	↓	Assie et al. (26)
	Microarray/RT-qPCR	17 ACC, 29 ACA	↓	Feinmesser et al. (61)
miR-214	TLDA	7 ACC, 19 ACA, 10 NA	↓	Tombol et al. (25, 62)
	NGS	45 ACC^a^, 3 NA	↓	Assie et al. (26)
	Microarray/RT-qPCR	17 ACC, 29 ACA	↓	Feinmesser et al. (61)
miR-375	TLDA	7 ACC, 19 ACA, 10 NA	↓	Tombol et al. (25, 62)
miR-511	TLDA	*id*.	↓	*id*.
miR-100	Microarray/RT-qPCR	10 ACC, 26 ACA, 21 NA	↓	Patterson et al. (58)
miR-125b	*id*.	*id*.	↓	*id*.
	Microarray/RT-qPCR	17 ACC, 29 ACA	↓	Feinmesser et al. (61)
miR-1974	Microarray/RT-qPCR	25 ACC, 43 ACA, 10 NA	↓	Ozata et al. (47)
miR-497	Microarray/RT-qPCR	*id*.	↓	*id*.
	NGS	45 ACC^a^, 3 NA	↓	Assie et al. (26)
	Microarray/RT-qPCR	17 ACC, 29 ACA	↓	Feinmesser et al. (61)
miR-139-3p	TLDA	4 ACC, 9 ACA, 4NA + validation cohort (*n* = 15)	↓	Schmitz et al. (63)
miR-675	TLDA	*id*.	↓	*id*.

*microRNAs highlighted in bold have been validated in several studies*.

*ACC, adrenocortical carcinoma; ACA, adrenocortical adenoma; NA, normal adrenal cortex*.

*↑ Upregulated, ↓ downregulated in ACC as compared to adenoma or normal adrenal cortices*.

*^a^The ACC group was composed of aggressive (poor prognosis) and non-aggressive (good prognosis) ACC*.

*^b^Overexpressed in aggressive versus non-aggressive ACC*.

*^c^Associated with shorter survival*.

**id*, *idem* refers to the line above*.

### miR-483-5p and miR-483-3p

miR-483-5p overexpression in ACC was consistently found in seven studies out of nine, whereas miR-483-3p overexpression was reported in five studies only (Table 1). Increased expression of miR-483-5p also identified a subgroup of patients that had significantly poorer prognosis (57). Upregulation of miR-483-5p was also observed in malignant pheochromocytomas as compared to benign tumors and associated with a poorer disease-free survival (64). miR-483 gene, which encodes both strands 5p and 3p, is located at 11p15.5 within the second intron of *IGF2* gene. The high expression of miR-483-5p observed in ACC was found to be correlated with the high expression of *IGF2* (58). However, the potential contribution of others mechanisms to miR-483-5p overexpression remains to be evaluated. Indeed, a functional β-catenin-dependent and *IGFI2*-independent-transcription start site located upstream of miR-483 locus has been reported in hepatocarcinoma and colon cancer cells (65). Oncogenic features of miR-483-5p and miR-483-3p have been suggested in Wilms’ tumors as well as in liver, breast, and colon cancers (66, 67). Veronese et al. further demonstrated that the oncogenic mechanism of miR-483-3p could be partially attributed to its ability to modulate the pro-apoptotic protein BBC3/PUMA, thereby protecting cells from apoptosis (66). Similar observations were subsequently made in ACC by Ozata et al. in the NCI-H295R ACC cell line (47). In the same study, downregulation of both miR-483-5p and miR-483-3p resulted in decreased proliferation. Using *in situ* hybridization, Wang et al. observed that miR-483-3p was overexpressed in 68% (17 of 25) of ACCs and in 12% (3 of 25) of ACAs (68). A combination of miR-483-3p and Smad4 expression improved the diagnostic accuracy provided by the Weiss score system. Interestingly, miR-483-3p but not miR-483-5p was found to be upregulated in childhood adrenocortical tumors (46). It is worth mentioning that overexpression of miR-483-5p or miR-483-3p in several human neoplasms suggests a wider involvement of this miRNA in human tumorigenesis. However, the dramatic increase of IGF2 and miR-483 expression in ACC (up to several hundreds of times as compared to ACA or NA) when compared to that of other cancers suggest a critical role for IGF2 locus in adrenocortical cancer development and progression. miR-483-5p but not miR-483-3p was recently shown to induce epithelial to mesenchymal transition (EMT) and to promote lung adenocarcinoma cell migration *in vitro* by targeting Rho GDP dissociation inhibitor alpha (RhoGDI1) and activated leukocyte cell adhesion molecule (ALCAM) (69). *In vivo*, miR-483-5p promotes lung adenocarcinoma metastases (69). Interestingly, IGF2 overexpression was not sufficient for tumor formation in transgenic mouse models (70–72). Along the same line, Veronese et al. showed that miR-483-3p inhibition could suppress tumorigenicity of HepG2 cells while no antitumor effect was elicited by inhibition of IGF2 (66). These results clearly indicate crucial oncogenic functions of miR-483 within *IGF2* gene and might explain why transgenic animals for IGF2 overexpression did not develop tumors as IGF2 transgenes were lacking miR-483 locus.

### miR-503

miR-503 was found significantly overexpressed in ACCs as compared to their normal and benign counterparts (26, 47, 61, 62) and in childhood adrenocortical tumors (46). Survival analysis indicated that high miR-503 was significantly associated with poor survival of ACC patients. In the study by Chabre et al., overexpression of miR-503 in ACC was observed in the discovery cohort but did not reach significance (59). miR-503 overexpression has been reported in retinoblastoma (73) as well as in parathyroid carcinoma (74). The role of miR-503 in ACC pathogenesis deserves further investigation. Indeed, miR-503 was reported as a tumor suppressor in several other cancers. miR-503 was found underexpressed in hepatocellular carcinoma (HCC) and was shown to inhibit angiogenesis *in vitro* and *in vivo* by downregulating expression of both fibroblast growth factor 2 (FGF2) and vascular growth factor A (VEGFA) (75). Low expression levels of miR-503 were associated with worse overall survival of HCC patients (76). Functional studies showed that miR-503 suppressed the proliferation of HCC cells by induction of G1 phase arrest through Rb-E2F signaling pathways (76). Furthermore, tumor suppressive effect of miR-503 was suggested in glioblastoma multiform (77). In this study, miR-503 was shown to exert its effect not only through suppression of cell proliferation by inducing G0/G1 cell cycle arrest and apoptosis but also through inhibition of cancer cell migration and tumor invasion. In addition, insulin-like growth factor-1 (IGF–1R) receptor mRNA was identified as a target of miR-503.

### miR-210

Overexpression of miR-210 in ACC has been observed in five studies out of eight (Table 1). High miR-210 was found associated with ACC aggressiveness and poor prognosis (60). miR-210 is a master miRNA in the cellular response to hypoxia (78). As hypoxia is a major hallmark of solid tumors, it is therefore not surprising that miR-210 is overexpressed in many tumors types. The expression of miR-210 is elevated in head and neck carcinoma (79), lung adenocarcinoma (80), late-stage small cell lung cancer (81), glioma (82), malignant melanoma (83), pancreatic ductal adenocarcinomas (84), ovarian cancer (85), and renal cancer (86). The stem–loop of miR-210 is located in an intron of a non-coding RNA on chromosome 11p15.5 (87). miR-210 is regulated by both HIF1α and HIF2α transcription factors (88, 89). However, Akt activation induces hypoxia-associated accumulation of miR-210 in a HIF-independent manner, suggesting that several signaling pathways can upregulate miR-210 in response to hypoxic stress (90).

### miR-195

miR-195 is also a major miRNA deregulated in ACCs (Table 1). miR-195 is significantly downregulated in ACCs compared to ACAs and its low expression in ACCs is significantly associated with poor overall survival (57). miR-195 levels are also significantly downregulated in childhood adrenocortical tumors (46). miR-195 gene is located on the chromosome 17p13.1 and is a member of the miR-15/16/195/424/497 family of miRNAs. Numerous studies have suggested that miR-195 promotes apoptosis while inhibiting cell proliferation. Restoration of miR-195 expression in the ACC cell line NCI-H295R impaired their proliferation *in vitro* (47). miR-195 is aberrantly expressed in multiple types of cancers, including human breast cancer (91), glioblastoma multiforme (92), gastric cancer (93), human HCC (94), and bladder cancer (95). Cyclin D1, CDK6, and E2F3 were identified as direct targets, suggesting that miR-195 plays a role in regulating G1/S transition. In colorectal cancer, miR-195 was shown to target Bcl-2 and thereby to inhibit tumorigenicity through apoptosis (96). In breast cancer, the methylation state of CpG islands upstream of the miR-195/497 gene was found to be responsible for the downregulation of both miRNAs (91). A forced expression of miR-195 or miR-497 suppressed breast cancer cell proliferation and invasion. In this study, Raf-1 and Cyclin D1 were identified as direct targets of miR-195. In addition, miR-195 expression in breast cancer was found to be inversely correlated with malignancy.

### miR-335

miR-335 was highly significantly downregulated in ACCs as compared to ACAs and normal adrenocortical tissue in several studies (Table 1). miR-335 has been shown to act as tumor suppressor or oncogene depending on cancer types. These findings suggest a tissue-specific role for miR-335. miR-335 is located at 7q32.2. It is downregulated in breast cancer (97, 98), while it is upregulated in colon cancer (99) and pediatric acute leukemia (100). The genetic deletion and epigenetic promoter hypermethylation occurring at miR-335 locus has been correlated with breast cancer metastases and ovarian cancer recurrence (98). In breast cancer, miR-335 suppresses metastasis and migration through targeting of the progenitor cell transcription factor SOX4 and extracellular matrix component tenascin C (101). More recently, miR-335 was shown to act as a tumor suppressor to regulate clear cell renal cell carcinoma cell proliferation and invasion through downregulation of BCL-W expression (102). Moreover, miR-335 suppresses breast cancer cell migration by negatively regulating the HGF/c-Met pathway (103).

### microRNAs Differentiating between Aggressive and Non-Aggressive ACC: The miR-506-514 and DLK1-MEG3 Clusters

Recent genomic studies led to the identification of two distinct molecular subgroups of ACC with different outcomes: the C1A group, associated with poor prognosis, and the C1B group, associated with better prognosis (21, 26). Using Illumina sequencing to determine miRNA expression in 45 ACC, Assie et al. identified three ACC clusters characterized by three distinct miRNA profiles Mi1, Mi2, and Mi3. Mi1 and Mi2 clusters belong to the C1B group, while the Mi3 cluster characterizes the C1A group. Strikingly, the Mi1 cluster displayed the largest differences in miRNA expression relative to NA samples. This group was characterized by upregulation of 11 miRNAs belonging to the miR-506-514 cluster located at Xq27.3. Interestingly, these observations were in agreement with those reported by Chabre et al. in a small cohort of ACC [discovery cohort: 6 aggressive ACC (aACC, poor prognosis), 6 non-aggressive ACC (naACC, good prognosis) and 6 ACA; validation cohort: 9 aACC, 9 naACC, and 10 ACA] (59). In this study, miR-508-3p, miR-509-3p, miR-513-3p, and miR-514, which belong to the miR-506-514 cluster, were also found upregulated in naACC as compared to aACC. An oncogenic role for the miR-506-514 cluster was reported in melanoma where these miRNAs promote not only melanoma progression but also melanocyte transformation (104). The mechanisms underlying the upregulation of this oncogenic miRNA cluster in non-aggressive ACCs (C1B group) then its downregulation in aggressive ACC (C1A group) remain to be determined. Along the same line, comparing the miRNAs related to melanoma early progression to those involved in metastasis, Mueller et al. identified miR-506 and miR-507 as upregulated during early progression and subsequently downregulated in metastatic colonization (105). One can speculate that the functions of sub-clusters of the miR-506-514 cluster versus the full miR-506-514 cluster support shifting roles for various members depending on the stage of ACC progression. Identifying downstream targets of the miR-506-514 cluster may reveal important pathways contributing to ACC pathogenesis.

In humans, the DLK1-DIO3 genomic region, located on human chromosome 14 (14q32) contains the paternally expressed imprinted genes DLK1, RTL1, and DIO3 and the maternally expressed imprinted genes MEG3 and MEG8, and antisense RTL1. This region hosts, in addition to the two long intergenic RNAs MEG3 and MEG8, one of the largest miRNA clusters in the genome, with 53 miRNAs in the forward strand and one in the reverse strand (106). Assie et al. found downregulation of 38 miRNAs belonging to the imprinted DLK1-MEG3 cluster located at 14q32.2 in the good prognosis group of ACC (Mi1 tumors) (26). LOH of chromosome arm 14q was detected in all Mi1 tumors, associated with full methylation of MEG3 promoter. In line with these data, Chabre et al. reported that several miRNAs belonging to the DLK1-MEG3 (miR-370, miR-376a, miR-376b, miR-376c, miR-377, miR-379, miR-382, miR-411, miR-487a, miR-494, and miR495) were downregulated in non-aggressive ACC as compared to aggressive ACC (59). Quantitative PCR analysis further confirmed that miR-376a, miR-376b, and miR-376c were significantly underexpressed in naACC. Using microarray expression and qRT-PCR assays, Teferedegne et al. found that increases in the expression of miR-376a correlated with the acquisition of tumorigenic phenotypes in cell lines of non-human primates (107). miR-376a overexpression was associated with nodal metastasis in the progression of gastric cancer (108). Interestingly, overexpression of the DLK1-MEG3 was positively correlated with HCC stem cell markers and associated with poor survival rate in HCC patients (109). In another study, overexpression of miR-376c in ovarian cancer cells was found to block cisplatin-induced cell death (110). The investigators suggested that miR-376c enhances proliferation, survival, and chemoresistance by targeting activin receptor-like kinase 7 (ALK7). The role of the DLK1-MEG3 cluster in ACC aggressiveness awaits further investigations.

### Deregulation of the miRNA-Processing Machinery in Adrenocortical Cancer

In addition to genomic or transcriptional alterations, deregulated miRNA expression can arise from failure in miRNA biogenesis. Several studies have shown that miRNA expression is globally suppressed in cancer cells compared with normal tissue, suggesting that miRNA biogenesis might be defective in cancer (39). A decreased expression of Dicer1 and Drosha has been reported in lung and ovarian cancers (111, 112). In addition, low Drosha or Dicer1 expression levels were associated with advanced tumor stage and poor clinical outcome in patients with ovarian cancer (112). On the contrary, Dicer1 overexpression was reported in melanomas (113) and was associated poor survival in colorectal cancer (114). Higher expression of Drosha was found in cervical squamous cell carcinomas (115) and epithelial skin cancers (116). Its overexpression was associated with poor prognosis in esophageal cancer (117). These variations of Dicer1 and Drosha expression levels among different tumor types suggest that miRNA-processing complexes act as tumor suppressors or oncogenes depending on cellular context. In adrenocortical cancer, two studies analyzed Tarbp2, Dicer1, and Drosha expression in ACA and ACC. Using RT-qPCR, Caramuta et al. reported a significant overexpression of Tarbp2, Dicer1, and Drosha transcripts in carcinomas compared with adenomas or NA cortices (43 ACA, 30 ACCs, and 9 NA cortices) (48). In addition, mRNA expression of Tarbp2, but not Dicer1 and Drosha could discriminate between ACAs and ACCs. Copy number gain of the *Tarbp2* gene was observed in 57% of the ACCs analyzed in this study. Inhibition of Tarbp2 expression in NCI-H295R cells resulted in a decreased cell proliferation and induction of apoptosis. Tarbp2 and Dicer1 were demonstrated as targets of miR-195 and miR-497, two miRNAs downregulated in ACC, suggesting that miRNAs might contribute to deregulation of their own biogenesis. de Sousa et al. analyzed Tarbp2 and Dicer1 expression in a cohort of 75 ACAs and 79 ACCs (118). Immunohistochemical analysis revealed that Dicer1 protein overexpression was found in 49% of ACCs and 32% of ACAs, while its mRNA was overexpressed in 60% of ACCs and 23% of ACAs. Nevertheless, the authors reported that metastatic ACC were characterized by a weak Dicer1 expression as compared to their non-metastatic counterparts. Furthermore, a weak Dicer1 expression was associated with reduced disease-free and overall survival. In contrast to Caramuta et al. study, no significant differences were found between ACCs and ACAs in terms of Tarbp2 protein or mRNA levels. The reasons for these discrepancies between the two studies remain unclear. They might be due to the size and the heterogeneity of the cohorts. Another regulator of miRNA biogenesis, LIN28, has been studied in adrenocortical tumors (119). LIN28 is an RNA-binding protein that binds to let-7 miRNA precursors (pri- and pre-let-7) and blocks their processing by Drosha in the nucleus and by Dicer in the cytoplasm (120). LIN28 was found underexpressed in aggressive ACC as compared to their non-aggressive counterparts (119). In the same study, Faria et al. reported that both weak expression of LIN28 and overexpression of miR-9, a negative regulator of LIN28, were associated with poor outcome of ACC patients. Nevertheless, a direct functional interaction between LIN28 and miR-9 was not investigated. When analyzing the global expression profile of miRNAs in the ACC cohort studied by Assie et al., it seems that miRNAs are rather overexpressed in the poor prognosis group as well as in a subpopulation of good prognosis group (26). Indeed, among the significantly deregulated miRNAs in ACC with poor outcome (C1A group), 86% were found upregulated and 14% were downregulated (Mi3 cluster). In the C1B group with good prognosis, 85% of the miRNAs were upregulated and 15% were downregulated in the Mi2 cluster, while only 45% were upregulated and 55% were downregulated in the Mi1 cluster. Chabre et al. also observed that all the discriminatory miRNAs between aggressive and non-aggressive ACC were upregulated in aggressive ACCs (59). Putting all these data together, it seems that the contribution of the miRNA-processing machinery disruption to the global deregulation of miRNA expression in ACC needs further clarifications.

## Circulating microRNAs as Potential Non-Invasive Diagnostic and Prognostic Biomarkers in ACC

Since the discovery of cell-free circulating miRNAs, numerous studies have reported that specific miRNA levels in body fluids reflect various disease states (42). Although the precise mechanism of miRNA release into the extracellular environment is not completely elucidated, some miRNAs are probably released as a result of normal or pathology-associated cell death (41, 121). Other cellular miRNAs were shown to be released into body fluids through active secretion. Notably, a ceramide-dependent secretory pathway that involves sphingomyelinase 2 has been described (122). Circulating miRNAs are either encapsulated in small vesicles that are referred to as microvesicles or exosomes depending on their size, or complexed to HDL and RNA-binding proteins. Nevertheless, Turchinovich et al. reported that most extracellular miRNAs in blood plasma and cell culture conditioned media are not associated with exosomes or microvesicles but are bound to Ago2, a component of the RISC complex (123). The role of HDL-mediated miRNA transport in the context of adrenocortical tumorigenesis deserves further investigations as HDL may also function as a source of ApoA1-dependent selective uptake of cholesterol in steroidogenic cells through the scavenger receptor SR-B1 (124). A potential connection between cholesterol uptake and miRNA internalization in adrenocortical cells remains an open and fascinating question.

The field of circulating miRNA research in ACC is emerging and we are still far from having a clear picture. The transfer to the clinic of circulating miRNA-based test requires the establishment and implementation of standardized operating procedures. Unspecific fluctuations of circulating miRNAs may arise upon different serum/plasma preparation methods, different storage conditions of samples, and the presence of hemolysis (125–127). Another major concern is the potential interference of the therapy with circulating miRNA levels that may confound the interpretation of the results. Prospective studies in which blood samples will be timely collected before and after treatment of ACC patients are needed. Three studies analyzed circulating miRNA levels in ACC patients (Table 2). All three studies reported an increase in miR-483-5p in ACC patients, which seems to accompany the previously identified increase of miR-483-5p in tumor tissue (59, 128, 129). However, there are substantial differences in the findings of these studies, which may be in part due to the different blood material used, i.e., serum or plasma and the normalization strategies. Chabre et al. spiked-in *C. elegans* cel-miR-39 not only to monitor the efficiency of RNA extraction but also to use it as a normalization miRNA. Based on the identification of deregulated levels of miR-195, miR-335, miR-139-5p, miR-376a, and miR-483-5p (Table 2), they assessed their potential diagnostic value. The most informative miRNA for the discrimination of ACA from ACC patients was miR-195 [area under curve (AUC) = 0.948, 95% CI: 0.819–0.994, *p* < 0.0001]. miR-195 could detect individuals with adrenocortical cancer with 90.9% sensitivity and 100% specificity. miR-335 and miR-376a were also good markers of malignancy with an AUC of 0.837, and 0.811, respectively. Although miR-139-5p displayed high sensitivity for the discrimination of ACA from ACC patients (87.5%), its specificity was moderate (65%, AUC = 0.714, *p* = 0.023). Importantly, miR-483-5p could distinguish non-aggressive ACC from aggressive ACC patients with 85.7% sensitivity and 100% specificity (AUC = 0.929, 95% CI: 0.741–0.994, *p* < 0.0001). Moreover, low levels of miR-195 and high levels of miR-483-5p were predictive of recurrence risk in ACC patients. Using plasma samples and endogenous miR-16 as a reference, Szabo et al. identified hsa-miR-100, hsa-miR-181b, hsa-miR-184, hsa-miR-210, and hsa-miR-483-5p miRNAs as significantly differentially expressed between ACA and ACC patient plasma samples (129). By combining endogenous hsa-miR-16 and spiked-in cel-miR-39, they found hsa-miR-181b and hsa-miR-483-5p as significantly differentially expressed. The dCT_hsa-miR-210_–dCT_hsa-miR-181b_ and the dCT_hsa-miR-100_/dCT_hsa-miR-181b_ pairs yielded the highest AUC values (0.87 and 0.85, respectively). In Patel’s study, it was found that the levels of miR-34a were increased in the serum of patients with ACC, while miR-34a was reported to be decreased in ACC tumors (128). Along the same line, Chabre et al. observed that miR-376a was significantly upregulated in ACC tumors, while it was significantly downregulated in the serum of patients with ACC. Opposite differential expression profiles of miRNAs in the circulation compared to parental cells are increasingly reported (130). These observations raise the question of an active mechanism by which selected miRNAs are promoted toward the extracellular space. Given the small cohorts used these studies, validation of circulating miRNAs as biomarkers for adrenocortical cancer requires an in-depth analysis in larger cohort of samples. Combinatorial use of multiple miRNAs should improve the sensitivity and specificity of biomarkers panels.

**Table 2:** **Deregulated circulating microRNAs in patients with adrenocortical cancer**.

Validated miRNA in ACC compared to ACA or NA	Sample type and cohort composition	Signature	Reference
**miR-483-5p**	Serum, 23 ACC, 14 ACA, 9 NA	↑^a^,^b^,^c^	Chabre et al. (59)
	Plasma, 13 ACC, 12 ACA	↑	Szabo et al. (51, 129)
	Serum, 17 ACC, 22 ACA	↑	Patel et al. (128)
miR-100	Plasma, 13 ACC, 12 ACA	↑	Szabo et al. (51, 129)
miR-181b			
miR-184			
miR-210			
miR-34a	Serum, 17 ACC, 22 ACA	↑	Patel et al. (128)
miR-195	Serum, 23 ACC, 14 ACA, 9 NA	↓^a^,^c^	Chabre et al. (59)
miR-335	Serum, 23 ACC, 14 ACA, 9 NA	↓^a^	Chabre et al. (59)

*ACC, adrenocortical carcinoma; ACA, adrenocortical adenoma; NA, normal adrenal cortex*.

*↑ Upregulated, ↓ downregulated in the serum or plasma of patients with ACC as compared to patients with adenoma or healthy subjects*.

*^a^The ACC group was composed of aggressive (poor prognosis) and non-aggressive (good prognosis) ACC*.

*^b^Overexpressed in the serum from patients with aggressive versus patients with non-aggressive ACC*.

*^c^Associated with shorter survival and recurrence risk*.

*Circulating microRNA levels were determined by RT-qPCR in the three cited studies*.

## Adrenocortical Cancer Cell-Derived Exosomes: Players in the Communication with the Tumor Microenvironment?

**Figure 3:**
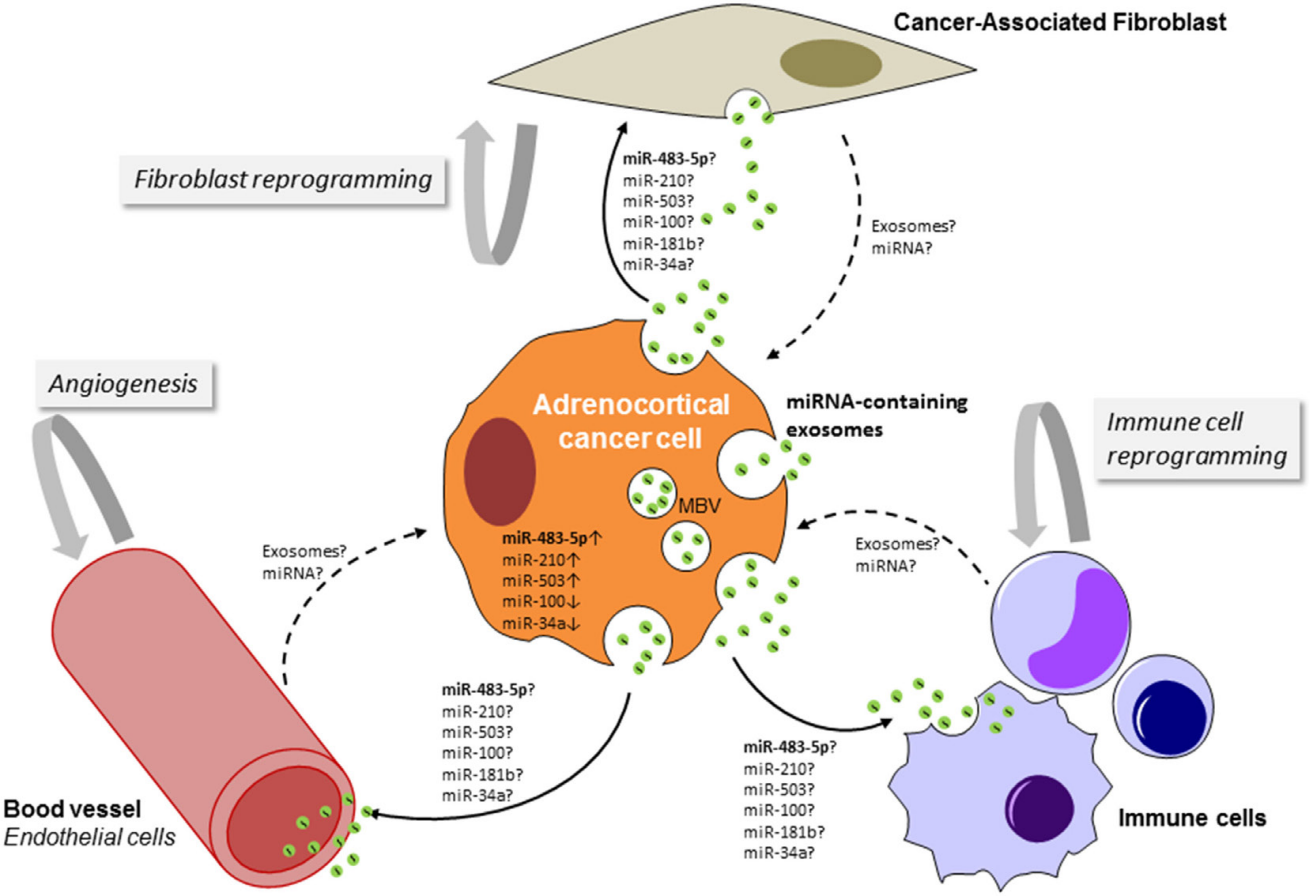
**Schematic representation of the potential cross-talk between adrenocortical cancer cells and cells of the tumor microenvironment (TME)**. By targeting cells of the TME, adrenocortical cancer cell-derived exosomes could favor stimulation of angiogenesis, production of pro-inflammatory cytokines, alterations of the extracellular matrix, and generation of a pre-metastatic niche. The TME-derived exosomes (dashed lines) could enhance growth and survival of cancer cells, promote invasion and induction of epithelial to mesenchymal transition, and also drug resistance. MBV, multivesicular body.

Although the release of apoptotic bodies during apoptosis has long been recognized (131), the fact that healthy cells also shed vesicles from their plasma membrane has only recently become appreciated. Numerous studies are beginning to decipher the molecular mechanisms of exosomes sorting and release. Notably, the content of cancer cell-derived exosome differs from exosomes derived from normal healthy cells and cancer cells have an increased rate of exosome release (132). The concept that exosomes are signaling entities in the cross-talk between various cell types is expanding (133). One can anticipate that exchange of exosomes between adrenocortical cancer cells and their neighboring components in the tumor microenvironment (TME), such as vascular endothelial cells, immune cells, and fibroblasts, might occur (Figure 3). The cellular origin of the multiple significantly deregulated miRNA in ACC tumor tissue as well as in the serum of the patients has not been deciphered so far. The expression profiles of miR-335, miR-195, miR-376a, miR-376b, miR-376c, miR-139-5p, and miR-483-5p in the NCI-H295R cell line were similar to their expression in the patients ACC samples, suggesting that their deregulation occurs in cancer cells (59). Increased circulating levels of miR-483-5p paralleled its marked upregulation in ACC. Nevertheless, defining the cellular localization of the other deregulated miRNA in ACC by performing *in situ* hybridization may help to unravel the potential interaction between ACC cancer cells and their surroundings and also the relationship between intratumoral and circulating miRNAs. Luga et al. reported a key role for cancer-associated fibroblast-derived exosomes in mobilizing autocrine Wnt-planar cell polarity (PCP) signaling in breast cancer cells to stimulate invasive behavior and metastasis in animal models (134). Transfer of exosomal miRNAs to endothelial cells has been shown to disrupt the vascular endothelial barrier by targeting the tight junction protein ZO-1 during early breast pre-metastatic niche formation (135). A seminal study performed by the group of Liberman demonstrated that exosomes released by metastatic cancer cells can transfer metastatic capabilities to non-metastatic cells. This transformation is directed by the miR-200 family that is known to mediate the mesenchymal-to-epithelial transition (136). The exchange of exosomal miR-21 and miR-155 between neuroblastoma cells and human monocytes has been implicated in the development of resistance to chemotherapy (137). All these observations open new perspectives in the field of exosome-mediated cell-to-cell communication within the TME in ACC.

## Silencing and Recovery of Altered microRNAs: A Future Therapeutic Approach in Adrenocortical Cancer

microRNAs are at the center of a complex combinatorial code regulating gene expression. Thus, identifying the relationships between miRNA signatures and adrenocortical cancer could help to understand the mechanisms behind the pathological processes and to develop therapeutic strategies. The biosynthesis, maturation, and activity of miRNAs can be manipulated by specific oligonucleotides that are complementary to mature miRNAs (138). Overexpression of miRNAs can be triggered by using synthetic miRNA mimics. Conversely, overexpressed miRNAs can be silenced by antagomiRs or miRNA sponges to restore miRNA balance in cancer networks (139). For example, inhibition of miR-21 and miR-17-92 was associated with reduced tumor growth, invasion, angiogenesis, and metastasis (140, 141). The therapeutic potential of miR-122 antagonist, miravirsen, in the treatment of Hepatitis C was evident from a multi-centric phase II trial (142). Although such findings are exciting, targeted miRNA therapeutics remain in the early stages of development and are essentially limited to *in vitro* and murine models of cancer. The development of relevant animal models of ACC is essential to the preclinical testing of miRNA-based therapies. On the other hand, though miRNAs possess tremendous therapeutic potential for cancer, a major concern remains their delivery system that may induce off-target effects. Lipid-based vehicles, viral systems, and cationic polymers are the main delivery tools for miRNA-based therapeutics (143). Each of these strategies has its own challenges and still needs improvements to address problems, such as cytotoxicity, immunogenicity, and low efficiency. Due to their natural role in miRNA secretion and shuttling between different cells, exosomes are of great interest in miRNA therapeutics. Their non-synthetic nature potentiates them for more efficient and non-immunogenic delivery of cargo while they maintain the cargo integrity and stability. Moreover, exosomal membranes contain proteins that have specific receptors on the surface of recipient cells. Therefore, they can selectively target cell types of interest and modifying their miRNA contents. Two delivery systems using liposome formulated miRNAs or miRNAs packaged in EnGeneIc Delivery Vehicles (EDVs) (144, 145) have reached the clinic and are currently under evaluation in cancer clinical trials. Recently, Glover et al. first reported that systemic administration of miR-7-containing EDV reduces ACC xenograft growth through the targeting of Raf-1 proto-oncogene and mechanistic target of rapamycin (mTOR) (49). This work is the first study investigating the therapeutic potential of miRNAs in ACC and many others should be expected.

## Conclusion

The discovery of miRNAs has considerably changed our understanding of gene regulation and new findings over the last decade have established that miRNA are key players in cancer molecular biology. Deregulations of miRNAs expression and activity are important steps in the development of many cancers, including adrenocortical cancer. On the basis of expression profiling of miRNA in ACC, several groups have identified miRNAs enabling diagnosis and prognosis of ACC. These findings need to be validated in larger cohorts and in prospective studies. Another important question for the management of ACC is the possibility of predicting patient response to therapy. Identification of specific miRNAs as significant indicators for response to mitotane or chemotherapy may guide the clinicians and provide an opportunity for personalized medicine. To improve our knowledge as to the role of miRNAs in ACC pathogenic pathways, functional effects of specific miRNAs need more comprehensive and thorough studies. The occurrence of miRNAs in the serum and plasma of ACC patients lays the groundwork for their development as minimally invasive biomarkers. The fact that miRNAs can function as cellular master regulators, show broad activity across multiple cancer types, and appear to specifically inhibit metastasis suggests that they could be used as therapeutic agents in cancers for which there are no or few treatment options, such as ACC. Nevertheless, a number of scientific and technical considerations must be addressed before we could reach these promising prospects.

## Author Contributions

NC conceived and wrote the manuscript.

## Conflict of Interest Statement

The author declares that the research was conducted in the absence of any commercial or financial relationships that could be construed as a potential conflict of interest.
